# EUS-guided choledochoduodenostomy as salvage therapy for surgical choledochoduodenostomy dysfunction

**DOI:** 10.1097/eus.0000000000000201

**Published:** 2026-05-15

**Authors:** Péter Lassú, István Szabó, Csaba Lőrinczi, Zsolt Dubóczki, Attila Doros, Richárd Szmola

**Affiliations:** 1 Division of Gastroenterology, Csolnoky Ferenc Hospital, Veszprém, Hungary; 2 Department of Radiology, National Institute of Oncology, Budapest, Hungary; 3 Interventional Endoscopy Unit, National Institute of Oncology, Budapest, Hungary; 4 Department of Visceral Surgery, National Institute of Oncology, Budapest, Hungary.

EUS-guided choledochoduodenostomy (EUS-CDS) offers definitive biliary drainage as a minimally invasive alternative to conventional percutaneous (percutaneous transhepatic biliary drainage), endoscopic (endoscopic retrograde cholangiopancreatography), or surgical approaches, with high success rates and low morbidity.^[1–3]^

A 72-year-old male patient has been operated on for a large (11 cm) retroperitoneal leiomyosarcoma. Painless jaundice developed in the first postoperative week, and urgent surgical choledochoduodenostomy was performed at the referring center. Adjuvant oncological treatment was initiated in our hospital, and follow-up imaging revealed novel liver metastases (segments 5 and 6) adjacent to the diffusely dilated biliary tree. Liver function tests were normal [Figure 1]. Percutaneous radiofrequency ablation (RFA) treatment of the isolated liver metastases (6 and 17 mm in size) was scheduled 10 months after the index surgical procedure. Because the risk of a biliary fistula formation was not negligible, we attempted pre-RFA biliary decompression through endoscopic retrograde cholangiopancreatography, but the dilated ducts were inaccessible via the papilla or stenosed surgical biliodigestive anastomosis [Figure 2]. After oncologically successful RFA treatment, a post-RFA biloma formed, necessitating a percutaneous biliary access: the surgical anastomosis could not be traversed, and an external percutaneous transhepatic biliary drainage was placed temporarily [Figure 3].

**Figure 1.**
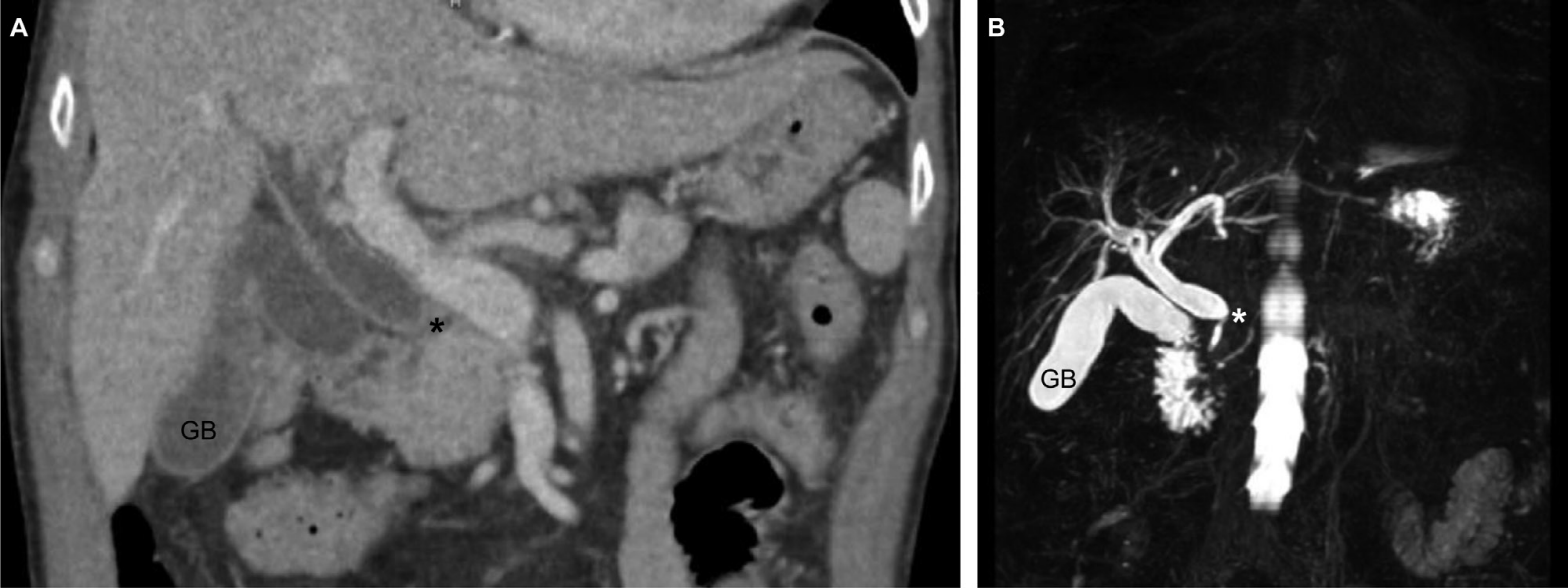
CT (A) and MRCP (B) images show a markedly dilated biliary tree above the surgical choledochoduodenostomy (*asterisks*). CT, computed tomography; MRCP, magnetic resonance cholangiopancreatography; GB, gallbladder.

**Figure 2.**
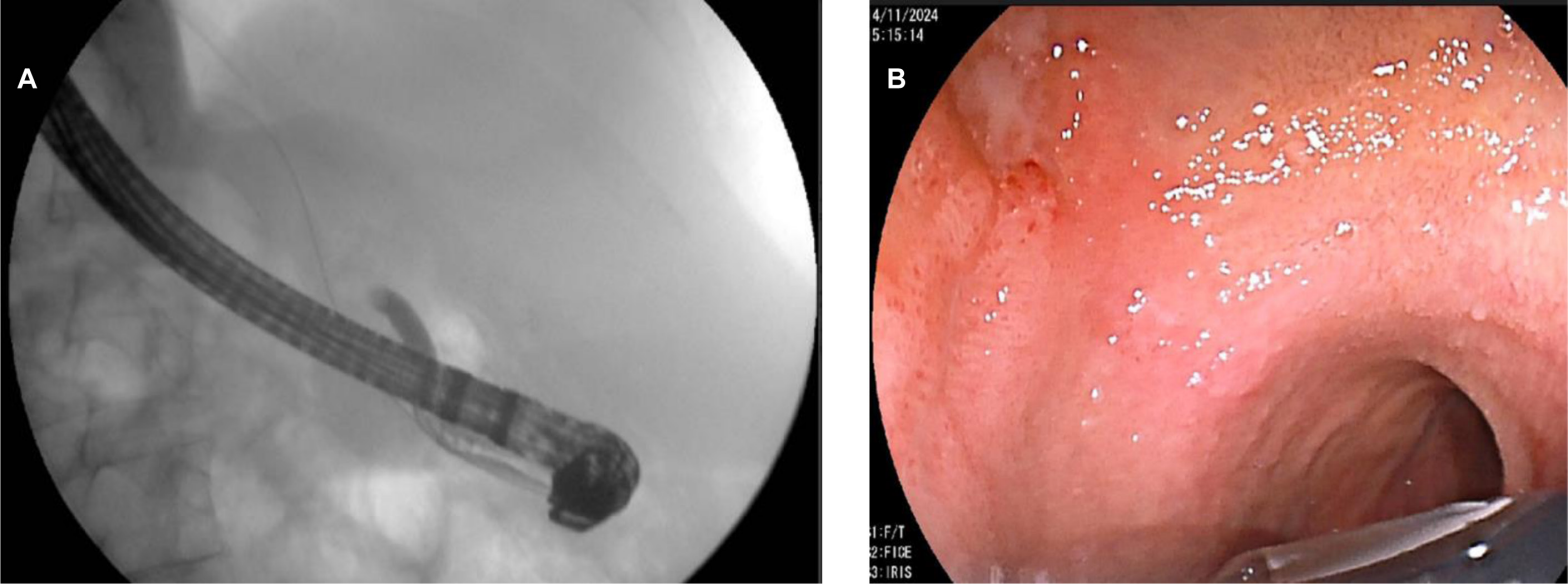
Dilated biliary tree inaccessible via the papilla (A, ERCP) or stenosed surgical biliodigestive anastomosis (B, endoscopic view). ERCP, endoscopic retrograde cholangiopancreatography.

**Figure 3.**
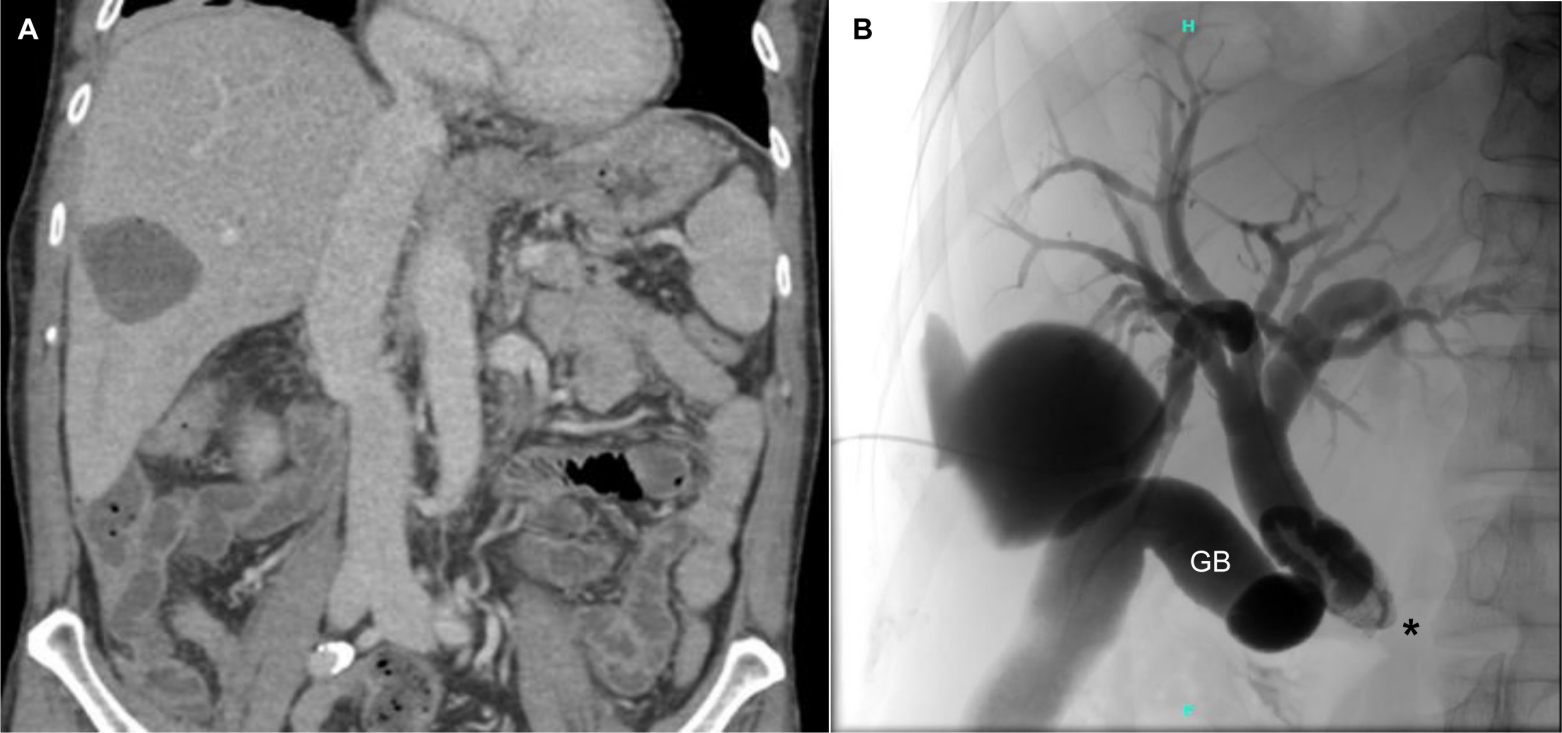
CT image shows a post-RFA biloma formation (A), during percutaneous biliary access the surgical anastomosis (*asterisk*) could not be traversed, an external PTD was placed temporarily (B). CT, computed tomography; GB, gallbladder; RFA, radiofrequency ablation; PTD, percutaneous transhepatic biliary drainage.

Finally, an EUS-CDS was attempted. The caliber of the common bile duct (CBD) was only 6 mm after percutaneous decompression; therefore, we first filled up the CBD lumen with saline through the external drain to have a 16-mm-wide CBD target [Figure 4], and placed a Hot AXIOS lumen-apposing metal stent (6 mm × 8 mm; Boston Scientific, Marlborough, MA, USA) using the free-hand technique^[4]^ successfully creating a choledocho-bulbar anastomosis [Figure 5]. No complications were observed in the post-interventional period; the percutaneous drain was removed shortly after creating the novel endoscopic CDS anastomosis. Follow-up computed tomography 6 months after the therapeutic EUS procedure showed a good EUS-CDS stent position; the patient is asymptomatic [Figure 6].

**Figure 4.**
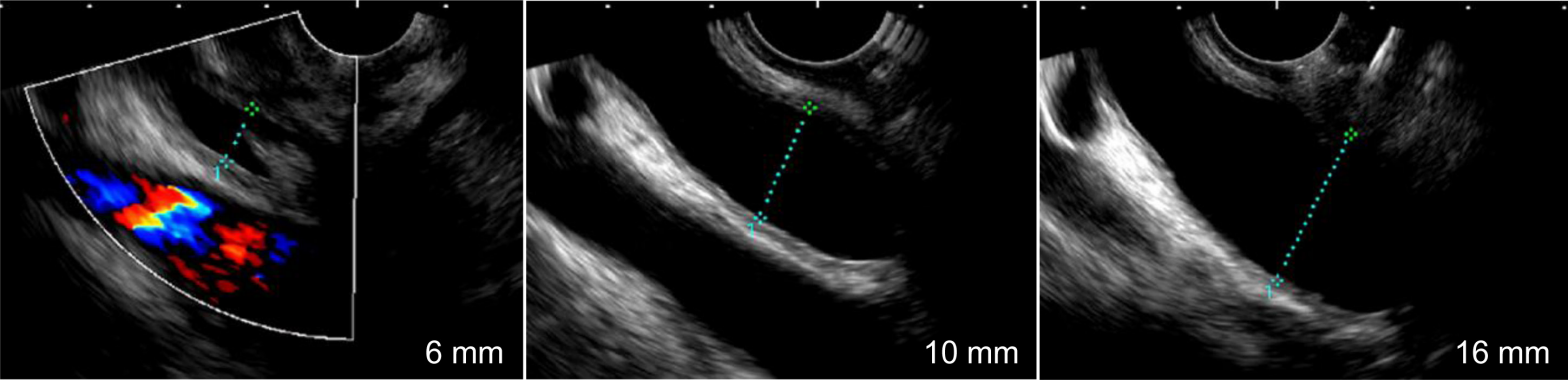
Common bile duct lumen gradually filled up through the external drain to reach a wide target for EUS-guided choledochoduodenostomy.

**Figure 5.**
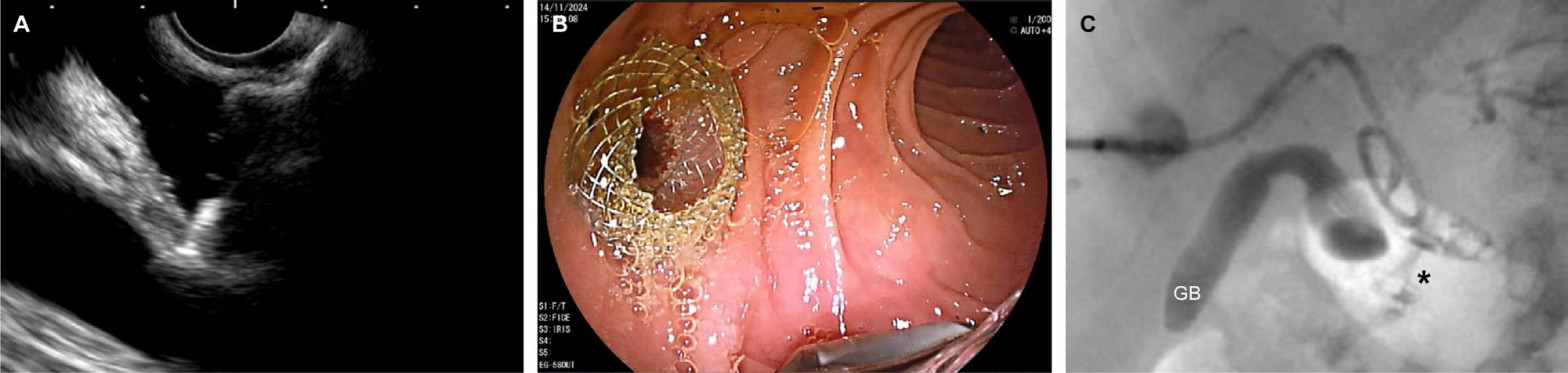
EUS-guided choledochoduodenostomy with lumen-apposing metal stent: distal flange opened on ultrasound view (A), proximal flange in good position on endoscopic view (B), confirmation of technical success on fluoroscopy (C). Asterisk shows position of the choledocho-bulbar metal stent; GB, gallbladder*.*

**Figure 6.**
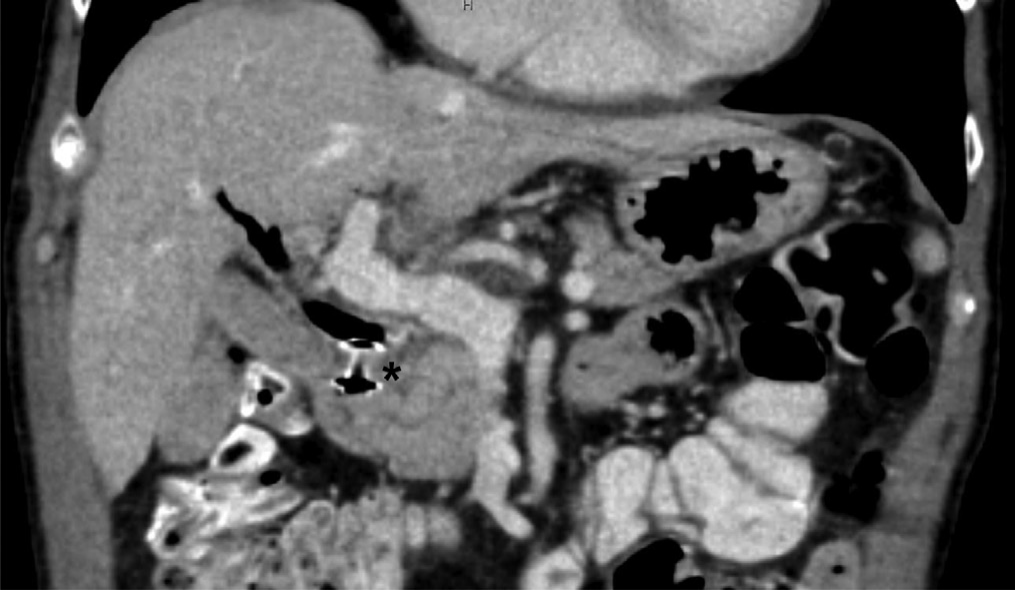
Follow-up CT shows good CDS stent position (*asterisk*) in the asymptomatic patient.

EUS-CDS is an effective drainage technique for complex biliary obstructions: surgically altered anatomy and non-dilated biliary ducts in our unique case. To the best of our knowledge, this is the first report of a “double CDS”: EUS-CDS salvage was performed after surgical CDS dysfunction, avoiding the morbidity associated with long-term external drainage or a repeated surgical intervention.

## Acknowledgment

We would like to thank Mária Egyedné Bihari and Gréta Sütő endoscopy nurses for excellent technical assistance.

## Ethical Statements

The case was conducted in accordance with the ethical standards described in the latest revision of the Declaration of Helsinki. Informed consent for patient participation and publication was received from the patient.

## Conflicts of Interest

The authors declare that they have no conflict of interest with regard to the content of this report.

## Author Contributions

All authors contributed to the study conception and design. Richárd Szmola performed the EUS-CDS intervention, percutaneous RFA and biliary access was performed by A. Doros. P. Lassú, C. Lőrinczi, and R. Szmola jointly wrote the manuscript. All authors approved the final version.

## Data Availability Statement

All data relevant to the case are included in the article.
